# From Another Stand-Point

**Published:** 1890-12

**Authors:** J. Allen Osman

**Affiliations:** Newark, N. J.


### FROM ANOTHER STAND-POINT.[1]

BY DR. J. ALLEN OSMAN, NEWARK, N. J.

Froude says, "The best we can do for one another is to exchange our thoughts freely, and that, after all, is but little." And I presume that no two persons can see the same thing in exactly the same light, even if viewed from the same stand-point; and it naturally follows that, from a different point of view, there will be a marked contrast in the ideas suggested.

Again, no two persons can have the same group of faculties developed in the same degree. For instance, an artist and engineer standing together, looking over the same scene, will have different impressions, although they view the same identical object. To the engineer the country presents itself in its practical aspect, and he sees, perhaps, a possible line of railroad, how to utilize some stream for a factory site, or the place to build a bridge; while the artist, of a more poetic nature, perceives the lights and shadows, the blending of vale and hill, tree and stream, and immediately conceives a picture of surpassing beauty. Hence our natural inclinations, our pet theories, or self-interest will warp our judgments and sway our imagination to such a degree that the results of our investigations will be far different.

It is well that this is so. Otherwise there would be but little

[1] Read before the New Jersey State Dental Society, July 16, 1890.

progress. If I were a minister, I should say that my text was "From Another Stand-Point," my theme "Dental Education," not, however, from the same stand-point of very many of the articles and discussions that have appeared from time to time, which have had for a central thought, raising the standard of qualification for entrance both to the college and the profession.

This subject has been so ably discussed in the past that there is but little to add; and yet I cannot pass the matter without alluding to one fact, and that is, while there has been marked attention paid to theory, perhaps the more practical has not received the attention it should.

There are certain mechanical laws that are immutable; they know no deviation, whether you apply them in building a bridge, erecting a building, constructing a locomotive, filling a tooth, or making and adjusting a denture,—in other words, the training of the eye and hand in mechanics.

This fact has been noticed by other members of the profession. I will cite two instances in support of my statement.

Once, while speaking to a gentleman in regard to an assistant he had just employed, on asking how he liked him, he replied, "He is a thorough gentleman, educated, and passed a brilliant examination; but, unfortunately, he doesn't know much about dentistry." He did not mean theoretically, oh, no; but practically. In other words, he was head-wise and not hand-wise.

This is about the experience of Dr. Shepard, president of the Massachusetts Board of Registration in Dentistry. In a paper read before the First District Dental Society of New York, and published in the *Dental Cosmos* of February, 1890, page 97, he uses this language: "And it may not be out of place for me here to make the confession that the certificate has, in some cases, been granted to graduates with great reluctance and mental reservation, and on account of considerations which were honorable, and of force to affect the decision of the Board; but which, we are convinced, should not in the future have the weight which they have had in the past;" and again, on page 99, I quote him as expressing the thought which I am endeavoring to bring out: "Dentistry, more than most vocations, forms a connecting link between the professions and trades, and must, in its highest development, exemplify each." In so far as its aims are benevolent, its investigations and studies confined to vital organs and functions, and its culture broad, liberal, and scientific, it is a profession. In so far as it deals with practical and mechanical operations, with the training of the hand to execute, and in much of the daily routine of the office, it is an art or trade. It approximates one or the other as it meets these tests; and I am sure that neither can be left out of the fully-rounded dentist. But I am as fully convinced that a knowledge and skill that will execute in a workman-like manner the various operations he is daily called upon to perform, is as essential, at least, as a knowledge of the metacarpal bones or the quadriceps extensors; and if he lack the former qualification he certainly will be a failure as a dentist, even if he possesses to a high degree the latter acquirement.

This, from the stand-point I am now occupying, seems one of the reforms called for in our education of dentists.

However, there is another duty for dentists to perform,—the dental education of the masses, the busy people. How to reach them, how to elevate them to a proper appreciation of the efforts put forth by the dental profession, for the correction of evils resulting from perverted nature and their own carelessness and ignorance concerning dental matters, is, I take it, a question worthy of our attention.

All operators will admit that the more intelligent the patient, all other things being equal, the greater and more satisfactory the results of the operation will be. I refer, of course, to dental intelligence; for it is well known that intelligence, education, and refinement along other lines have no correspondence with dental knowledge, for we have often found the most intelligent and well-informed persons in other respects wofully deficient in this direction. And this could hardly be otherwise when we consider how meagre the popular instruction has been in these matters; not but what the dentist would gladly answer any questions in this direction, but the nature of his work, requiring the closest attention, and the fact that his day only lasts while daylight is with him, prevents much instruction which would be gladly given if it were otherwise.

In many other arts and sciences there is a vast amount of popular instruction shed abroad by the press, both daily and weekly, secular and religious. Take, for instance, electricity. How much has been written on this subject! and there is scarcely an intelligent person but what has a fair idea of the wonderful inventions of various kinds that have been brought out; and while they are not electricians, they are in an appreciative mood to receive all that can come from these devices. Or, how often, in the press, do you see short pithy articles calling attention to the care of the eyes or ears, to diet, or the beneficial results of exercise and bathing, the importance of sleep, hygiene in its various manifestations, and, in fact, to a number of the ills that flesh is heir to!

It does not necessarily follow that a person can become his own physician, but it makes the patient a co-laborer to the common end of recovery, should he have any of these diseases.

But it is a rare thing to see any reference to the care of the teeth and the hygiene of the mouth. Is it because the dental profession is barren of knowledge that the people ought to have? I think not.

There has been some realization of this need, and a few efforts have been made to meet the demand, but it has been inadequate; some from not having been put in proper shape, and some from being too lengthy and cumbersome. The day of long, dry essays has gone by. The people are too busy. A book of twenty-five or fifty pages, or more, will in many cases lie on the shelf and become covered with dust. What is said must be put in a few words and in a palatable form. How can this be done?

Dental societies—State societies in particular—have an office to fulfil besides the one of mutual helpfulness, the discussion of technical and scientific subjects pertaining to their vocation. They must be the educators of the public.

They have, in a large measure, been on the alert to prohibit incompetent persons from inflicting the results of their ignorance on the public, and to the various State societies we owe the restrictive laws that are now in force in so many of the States; and while many of them are defective, some, perhaps, being too strict, others not enough so, time will probably bring about a law in all the States that shall be alike in its requirements and its penalties. Until this is done there is a weakness in dental legislation.

All this, however, has been along the line of self-preservation. There is a wider and higher mission for us to fulfil ere the harvest is ripe.

If it be true that people perish for lack of knowledge, it is equally true that people suffer misfortune and misery from the lack of dental knowledge; and it is a high and honorable opportunity that should be improved in scattering abroad among the masses such knowledge as will be of benefit to them. To prove that the public is hungry for such matters, I will mention an instance in our own city.

Some months ago one or, at most, two articles were printed in one of our papers giving a brief history of the advantages of the deposit plate; and to this day persons speak of it, asking about its merits, etc. "Straws show how the current tends."

As an example, if Dr. ———, located in a city or large town where two or more dentists were in practice, should interest himself and issue a pamphlet, or take occasion to deliver a few lectures in the schools before the scholars on the care of the mouth and teeth, he would at once arouse jealousies and bring down upon his head criticisms that would not be pleasant to bear.

If this picture is not overdrawn, then there is a field for dental societies; for any such step taken by and under the authority of such societies, with the absence of all names, would have greater weight. We need writers to prepare short and pithy articles on dental matters for the press.

The press is always ready and anxious to diffuse knowledge, but does not care for long and dry articles.

Then, again, the public should receive instruction in the hygiene of the mouth,—that cleanliness is indeed next to godliness. There are many things inherited from ancestors besides hereditary diseases, one of which is the neglect to realize the importance of frequent cleansing of the teeth, and perhaps a little knowledge in this direction will also tend to stir up some members of the dental profession. I had a patient the other day who informed me that his former dentist did not believe in removing tartar from the teeth, and said it was an unnecessary operation; that it insured them from decay.

Also, dentistry has passed the line where it is to be regarded as a luxury. It has become a necessity. Why do we not hear of the great expense of clothing, shoes, etc.? Because the people are educated that these things must be had, and they are replenished, when needed, in such manner as the funds will allow, and it must be so with the care of the teeth, and when the lesson is learned, and frequent examinations are made, then will people commence to realize the great benefits that dentistry is continually bestowing.

Another point that can be brought out with benefit to all concerned. It behooves the public to beware of that professional man who habitually absents himself from dental societies and from all the avenues of information. The stream never rises higher than its source, and when the source is low, then we cannot expect much. In other words, as Dr. Atkinson would say, the people must learn to differentiate; and if the lesson is learned, it will do as much to elevate the dental profession as any other one thing can possibly do; for the more intelligent the congregation the higher the acquirements of the preacher must be to serve them.

This law holds good in our profession as in the other. I do not think it is in the province of this paper to outline the details of what should be taught, or rather illuminated by the press, only perhaps to say in this connection that good results will be obtained by some method that brings it before the grammar department of the schools.

Psychologists tell us that when men and women pass a certain age, they fail to grasp new ideas; and then, again, to wait until adult life is to have the mischief accomplished. How much better results could be obtained, and how much more the services of the dentist would be appreciated if the masses of the people had only a glimmer of light on such things as are of daily practice; that it is not necessary to extract a tooth because it has had a so-called gumboil; that a tooth can ache when the "nerve is killed;" that the molar teeth do not come in again when taken out; that children do have teeth when they have not had others taken out; that a double row of teeth is not necessarily a "freak of nature." But why prolong the enumeration of these things? They suggest themselves to our minds.

Then again, what a field for enlightenment in the direction of the relation that should exist between the profession and the patient. The time is now ripe for a consultation fee, for many an hour is wasted by persons coming into a dental office and calling the dentist from his chair, for examination and consultation regarding what is best to be done. Perhaps it is a case of regulating; and when the advice is given, and the patient gone, a half-hour has been given away. This is simply because it is not customary for dentists to charge for such advice. Our brother physicians would receive a fee for such service, and it would be expected that he would.

There is not enough publicity given to the proceedings of the dental societies. They are, it is true, published for the education and profit of the profession; but there is much that ought to be wide-spread among the laity, and there is no one that is as capable of doing this as the different dental editors. They ought to have the experience to know what to give out and what to retain, and the dissemination of such facts as the wonderful advances made in the treatment and preservation of the teeth, the various methods of replacement now in vogue, the merits and elements of the various kinds of filling, what can be expected from pulpless teeth, and an infinite number of subjects would, if thoroughly understood, make the patient an appreciative one, and take better care of their own mouth and their children's.

I believe one thing thoroughly, that when trying to get the people to absorb matters of this kind, one subject only should be brought to notice at any one time, for when a number are treated the force is lost and the lesson is not remembered; hence my plea for the short, terse, and pithy articles for the press.

I will not trespass on your time and patience longer, but I feel that there is indeed a wide and inviting field in this direction, and that the public should have more light in these matters, and I as firmly believe that no profession has put forth greater efforts, nor has any profession reason to feel more gratified over its transcendent achievements.

I also realize that efforts put forth in educating the masses will be like the sower in the parable. Some seed will fall by the wayside and some be doomed by selfishness and forgetfulness; and then, too, some will be like "bread cast upon the waters," and after many days it will return.

